# THE IMPORTANCE OF COLONOSCOPY IN INFLAMMATORY BOWEL DISEASES

**DOI:** 10.1590/0102-672020180001e1374

**Published:** 2018-07-02

**Authors:** Márcio Alexandre Terra PASSOS, Fernanda Correa CHAVES, Nilson CHAVES-JUNIOR

**Affiliations:** 1 Hospital Universitário de Vassouras, Universidade Severino Sombra, Vassouras, RJ, Brazil

**Keywords:** Inflammatory bowel disease, Endoscopy, Colonoscopy, Crohn’s disease, Ulcerative colitis, Doença inflamatória intestinal, Endoscopia, Colonoscopia, Doença de Crohn, Colite ulcerativa.

## Abstract

***Introduction*:**

Endoscopic evaluation, particularly the macroscopic mucosal and histological results of ileocolic biopsies, is essential for the management of inflammatory bowel disease. Endoscopic appearance is not always sufficient to differentiate Crohn’s disease and ulcerative colitis, but there are some characteristics that favor one or another diagnosis. Both diseases have an increased incidence of colorectal carcinoma; so, surveillance colonoscopy is important for detecting early neoplastic lesions.

***Objective*:**

To update the importance of endoscopy in the evaluation, diagnosis and prognosis of inflammatory bowel disease.

***Method*:**

Search was done in the scientific literature of the TRIP database, chosen from clinical questions (PICO) with the following descriptors: “inflammatory bowel disease”, “endoscopy/colonoscopy”, “Crohn’s disease”, “ulcerative colitis” and “diagnosis/treatment”.

***Results*:**

Endoscopic investigation in patients with chronic colitis is quite accurate for the differential diagnosis between ulcerative colitis and Crohn’s disease. Endoscopy is indicated for ulcerative colitis during severe crisis due to its prognostic value. Another accepted indication for endoscopy in inflammatory bowel disease is its use in the screening for dysplastic lesion.

***Conclusion*:**

Ileocolonoscopy allows an accurate diagnosis of Crohn’s disease or ulcerative colitis in up to 90% of cases. The healing of the mucosa assessed by endoscopy after treatments despite not being consensus is still the gold-standard in the evaluation of remission of the disease. Colonoscopy is essential for long-term cancer surveillance and in the future the implementation of Confocal Laser Endomicroscopy seems to be very promising in assessing the initial dysplasia.

## RESUMO

***Introdução*:**

A avaliação endoscópica, em particular da macroscopia das mucosas e resultados histológicos de biópsias ileocolônicas, é essencial para o manuseio da doença inflamatória intestinal. A aparência endoscópica nem sempre é suficiente para diferenciar doença de Crohn e colite ulcerativa, porém existem algumas características que favorecem um ou outro diagnóstico. Ambas as doenças apresentam um aumento de incidência de carcinoma colorretal, assim, a colonoscopia de vigilância é importante para detectar lesões neoplásicas iniciais.

***Objetivo*:**

Revisar o papel da endoscopia na avaliação, diagnóstico e prognóstico de doença inflamatória intestinal.

***Método*:**

Foram pesquisados artigos científicos do banco de dados TRIP, escolhidos a partir de questões clínicas (PICO) com os seguintes descritores: “doença inflamatória intestinal”, “endoscopia/colonoscopia”, “doença de Crohn/colite ulcerativa” e “diagnóstico/tratamento”.

A investigação endoscópica em pacientes com colite crônica é bastante precisa para o diagnóstico diferencial entre colite ulcerativa e doença de Crohn. Ela é indicada na colite ulcerativa durante as crises graves de doença, por seu valor prognóstico. Outra indicação aceita na doença inflamatória intestinal é a sua utilização no rastreio da lesão displásica***.***

***Conclusão*:**

A Ileocolonoscopia permite diagnóstico preciso da doença de Crohn ou colite ulcerativa em até cerca de 90% dos casos. A cicatrização da mucosa avaliado por endoscopia após tratamentos, apesar de não ser consenso, ainda é o padrão-ouro na avaliação da remissão da doença.Colonoscopia é essencial para a vigilância do câncer em longo prazo. Futuramente a realização de Confocal Laser Endomicroscopy parece ser promissora na avaliação da displasia inicial.

## INTRODUCTION

Endoscopic evaluation, in particular mucosal and histological results of ileocolonic biopsies, is essential for the diagnosis of inflammatory bowel disease (IBD)^10^ ^,^ ^11^. Initial endoscopic examination should thoroughly describe the characteristics of the lesions observed and must include the visualization of the terminal ileum, always remembering that colonoscopies performed in the course of some type of treatment may obscure the characteristics of the disease for differential diagnosis^12,17,19^ .

The collection of material for histopathological analysis of all segments is mandatory, including macroscopically normal ones^1^ ^,^ ^7^ ^,^ ^17^ ^,^ ^21^ ^,^ ^24^ ^,^ ^27^.

The endoscopic appearance of IBD is not always sufficient to differentiate Crohn’s disease (CD) and ulcerative colitis (UC); however, there are some characteristics that favor one or the other diagnosis^16^. Both present an increased incidence of colorectal carcinoma. Thus, surveillance colonoscopy is important in detecting early-onset neoplastic lesions^4^.

The objective of this study was to review the role of endoscopy in the evaluation, diagnosis and prognosis of IBD.

## METHOD

The search for scientific articles was done through using the TRIP database (www.tripdatabase.com). The TRIP categories are: evidence-based synopses; clinical issues; systematic reviews; guidelines (North America, Europe, others); basic core research; eTextbooks; clinical trials; and general medical journals recovered from Medline (PubMed).

The chosen descriptors were from clinical questions (PICO). P (population) - inflammatory bowel disease; I (intervention) role of endoscopy/colonoscopy; C (comparison) Crohn’s/ulcerative colitis; O (outcome) - diagnosis/management

The studies were selected according to their relevancy and strength of evidence.

## RESULTS

The selected articles analyzed the role of digestive endoscopy in IBD, in relation to: diagnosis, prognosis, cancer surveillance, therapy and in relation to perspectives8,15,17.

Endoscopic investigation in patients with chronic colitis is quite accurate for the differential diagnosis between UC and CD17.

In a prospective study by Pera et al.^8^ ^,^ ^22^ 606 colonoscopies were performed in 357 patients with IBD and a precise diagnosis was demonstrated in 89% of the cases, whereas in 7% the diagnosis was undetermined8,14 and in 4% there was an error in the diagnosis. The misdiagnoses were more frequent (9%) in the subgroup of patients where endoscopy was performed during a severe outbreak of disease.

The combination of different endoscopic features, most suggestive of CD or UC11, was considered for an endoscopic diagnostic evaluation (CD was more likely if values ​​were greater than 4, whereas UC was more likely if the score was 4 or less, Table 1).

**TABLE 1:** Endoscopic diagnostic score for the differentiation between Crohn’s disease (4>) and ulcerative colitis (≤4)

ENDOSCOPIC CHARACTERISTICS	SCORE
Probable Crohn’s disease	
Segmental involvement of the mucosa	+55
Appearance in paving stone	+8
Aphthous ulcers / Serpentious / Linear ulcers	+4
Large deep ulcers	+4
Spared rectum	+5
Anal lesions	+15
Probable ulcerative colitis	
Continuous mucosal involvement	-2
Granular mucosal appearance	-3
Loss of vascular pattern	-2
Erosion	-7
Rectal involvement	-2

From M. Daperno, R. Sostegni, A. Lavagna, L. Crocellà, E. Ercole, C. Rigazio, R. rocca, A. pera. The role of endoscopy in inflammatory bowel disease. European Review for Medical and Pharmacological Sciences14

Endoscopy is indicated in UC during severe disease crisis due to its prognostic value^8^ ^,^ ^17^. Endoscopic examination should be performed without bowel cleansing, with minimal or no insufflation, for the potential risk of perforation. Carbonnel et al.^3^ ^,^ ^8^ demonstrated that total colonoscopy is feasible in 86% of severe cases of UC (73/85), and when severe endoscopic lesions (Table 2) are present, colectomy is very likely to be indicated: only 3/46 patients with severe endoscopic lesions (7%) compared to 29/39 patients without such lesions (74%) maintained their colon after treatment.

**TABLE 2:** Definition of severe lesions according to Carbonnell et al^3^ ^,^ ^8^

Severe endoscopic lesions	Moderate endoscopic lesions
Large deep ulcers	Erythematous mucosa
Loss of mucous layer (with or without islands of the mucous membranes)	Superficial ulcers
‘Well-like’ ulcers	Deep ulcers involving less than 10% of the surface
Large excoriations of the mucosa	

From M. Daperno, R. Sostegni, A. Lavagna, L. Crocellà, E. Ercole, C. Rigazio, R. rocca, A. pera. The role of endoscopy in inflammatory bowel disease. European Review for Medical and Pharmacological Sciences14

Daperno et al^8^ carried out a study for the creation and validation of a simplified endoscopic score for CD evaluation. For the construction of this score, four variables were considered: ulcers, proportion of surface covered by ulcers, proportion of surface covered by other lesions and stenoses. Each variable was scored from 0 to 3 on each follow-up. Ulcers were classified according to their size (diameter 0.1-0.5 cm, 0.5-2 cm, or >2 cm); the proportion of the ulcerated surface was expressed as a percentage (<10%, 10-30%, or >30%) as well as the proportion of the extent of the affected surface (<50%, 50-75%, or >75%). The stenoses were evaluated by the number and the possibility of being passed or not by the colonoscope (Table 3). This score is simpler and has a good correlation with “Crohn’s disease index of severity” and low variability among observers.

**TABLE 3:** Definitions of simplified endoscopic score for Crohn’s disease

VARIABLE	Score 0	Score 1	Score 2	Score 3
Size of ulcers	-	Aphthous ulcers 0,1 - 0,5 cm	Large ulcers 0,5 - 2 cm	Bigger ulcers >2 cm
Ulcerated surface	-	<10%	10-30	>30%
Surface affected	-	<50%	50-75%	>75%
Presence of stenoses	-	Single, can be overtaked	Multiple, can be overtaked	Cannot be overtaked

From Flores C. Projeto Diretrizes. Brazilian Society of Digestive Endoscopy 2008-201030

In UC, endoscopic findings are important to define the degree of disease activity. Endoscopy is the most objective method for assessing and quantifying damage to the colon mucosa. Several clinical and endoscopic indexes of UC activity were developed, but most were not validated. There are instruments for measuring UC activity based on clinical and endoscopic parameters or indexes that use the combination of this information. There is a significant variation of definitions and endoscopic parameters considered: friability (spontaneous bleeding or touching of the device), edema, enantema, loss of vascular pattern, mucosal granularity, presence of erosions and ulcerations. The most commonly used in clinical trials is the Mayo Clinic activity score (Table 4).

**TABLE 4:** Mayo Clinic Score

1. Frequency of bowel movements	2. Rectal bleeding
0=Normal to patient	0=bloodless
1=1-2 bowel movements / day>normal	1=blood streaks <½ of the time.
2=3-4 bowel movements / day >normal	2=live blood evident in most bowel movements
3=≥5 bowel movements / day >normal	3=evacuations with pure blood
3. Endoscopic findings	
0 = normal or inactive	
1=mild disease (enantema, loss of vascular pattern, slight friability).	
2=moderate disease (evident enantema, loss of vascular pattern, friability, erosions)	
3=severe illness (spontaneous bleeding, ulcerations).	
4. Overall medical assessment *	
0=normal	
1=mild disease	
2=moderate disease	
3=serious illness	
Score	Disease severity
≤2 no subscore >1	Clinical remission
3-5	Light activity
6-10	Moderate activity
11-12	Severe activity

From Flores C. Projeto Diretrizes. Brazilian Society of Digestive Endoscopy 2008-201030; *Global medical assessment takes into account the patient’s daily complaint of abdominal discomfort, overall feeling of well-being, physical examination findings, and patient performance for daily activities

### Endoscopy in cancer surveillance

Another accepted indication for endoscopy in inflammatory bowel disease is its use in the screening of dysplastic lesion^5^ ^,^ ^6^ ^,^ ^17^.

The guidelines of the American Society for Gastrointestinal Endoscopy^17^ indicate a colonoscopy every three years during the second decade of the disease, every two during the third decade, and annually in case of pancolitis.

Regarding chromoscopy Subramanian et al.^28^ in a meta-analysis concluded that chromoendoscopy was significantly better than white light endoscopy in the detection of dysplasia in patients with inflammatory bowel disease of the colon.

### Digestive endoscopy therapy

They are indicated in the complications of IBD. Endoscopic dilation of CD stenoses, when present, is performed by pneumatic balloon. The exact role of this procedure and of it in anastomoses is not clearly defined.

Shen et al.^26^ studied a total of 150 patients with post-ileostomy pouch stenosis due to complicated IBD. The sites were stenosis in the pouch entrance (n=96), outlet (n=73), afferent loop (n=33), and pouch body (n=2). A total of 406 therapeutic endoscopies were performed, with two perforations (0.46%) and four hemorrhages requiring blood transfusion (0.98%). The stock retention rates at 5, 10 and 25 years were 97%, 90.6% and 85.9%, respectively. At a mean follow-up of 9.6 (IQR 6-17) years, 131 patients (87.3%) were able to keep their pouches.

### Perspectives of endoscopy in IBD

Although colonoscopy is essential for the management of IBD, evaluation of most of the small intestine is not accessible to classical endoscopy; other endoscopic techniques are being performed, especially in the evaluation of the small intestine, which are double-balloon (or single) enteroscopy and the endoscopic capsule^13^ ^,^ ^20^ ^,^ ^23^.

Rahman et al.^23^ analyzed 98 patients submitted to double balloon enteroscopy performed in 81 patients (38 with known CD and 43 with suspicion of CD). The diagnostic yield was 87% (33/38 patients). The impact on clinical conduction was 82% (31/38). Common indications for enteroscopy with double balloon in patients with suspected CD were abnormal endoscopic capsule or other image. The diagnostic yield was 79% (34/43 patients). The impact on subsequent conduct was 77% (33/43). In 17% of patients (14/81), enteroscopy with double balloon failed to reach the lesion. There was a perforation.

The introduction of endoscopy capsule in 2000 revolutionized the ability to visualize parts of the small intestine mucosa not classically achieved by the conventional endoscope. The advantages of it include its non-invasive character and its ability to view proximal and distal parts of the intestine, while important disadvantages include the inability of the tissue sampling procedure and significant rate of incomplete examinations. The greatest limitation is the prohibited use in cases of known or suspected stenosis of the intestinal lumen due to the high risk of retention. Endoscopy capsule plays an important role in the early recognition of recurrence in patients with CD who have undergone resection of the ileum for the treatment of complications and in the management of patients and planning of therapeutic strategy^20^ ^,^ ^30^.

The Chang-Qing Li^18^ Confocal Laser Endomicroscopy (CLE) study with 73 patients showed that the evaluation of crypt and fluorescein architecture with this method showed good correlations with histological results (p<0.001). CLE seems to be more accurate than conventional white light endoscopy to assess mucosa. More than half of the patients with normal mucosa seen in conventional white light endoscopy presented acute inflammation in histology, whereas none with normal mucosa or with chronic inflammation seen in CLE showed acute inflammation in histology. Evaluation of microvascular changes by this method showed a good correlation with the histological findings (p<0.001). In the objective evaluation after CLE, subjective architectural classifications were supported by the number of crypts per image (p<0.001). A similar result was obtained by Francesca Salvatori et al.^25^

## DISCUSSION

Colonoscopy with ileoscopy allows direct visualization and biopsy of the mucosa of the rectum, colon and terminal ileum, and should be performed during the initial evaluation of patients with a clinical picture suggestive of IBD^17^.

The acquisition of detailed information and the colonoscopic score is important for the differential diagnosis of CD and UC^8^ ^,^ ^11^.

Documentation of endoscopic mucosal healing has become a critical component of outcome measurement. Although several studies show a disagreement between clinical and endoscopic remission, assessment of mucosal healing during IBD therapy is relevant to clinical practice, since it is considered the gold standard for a complete therapeutic response^7,8,11,14,16 ,17.22^.

Enteroscopy has a limited role in the management of patients with IBD, but allows macroscopic and histological evaluation, as well as providing therapeutic intervention^23^.

The endoscopic capsule enables direct and minimally invasive visualization of the mucosa of the small intestine. It may help to identify superficial lesions not detected by endoscopy and traditional radiography. It may be useful for the initial diagnosis of CD, for the detection of recurrences, to determine the extent of the disease, to evaluate the response to therapy, and for the differentiation of undetermined UC or CD^17^ ^,^ ^20^ ^,^ ^30^.

Individuals with long-standing IBD and especially extensive UC have an increased risk for the development of colorectal dysplasia and should undergo colonoscopy surveillance. Chromoendoscopy may improve sensitivity during colonoscopic surveillance by allowing targeted biopsies of increased mucosal change. Although promising, chromoscopy has not yet been adopted in routine practice.

The CLE study seems to be very promising in evaluating the inflammatory process of the mucosa, especially in macroscopically normal and dysplasia evaluation^17^ ^,^ ^18^ ^,^ ^25^ ^,^ ^26^.

## CONCLUSION

The ileocolonoscopy allows accurate diagnosis of CD or UC in about 90% of the cases. The healing of the mucosa evaluated with endoscopy after treatment is still the gold standard in the evaluation of remission of the disease. Finally, it is essential for long-term cancer surveillance and in the future the implementation of Confocal Laser Endomicroscopy (CLE) seems to be promising in evaluating the inflammatory process of the mucosa and dysplastic alteration. The endoscopic therapeutic management of stenoses can be employed more frequently minimizing the need for surgical procedures.
